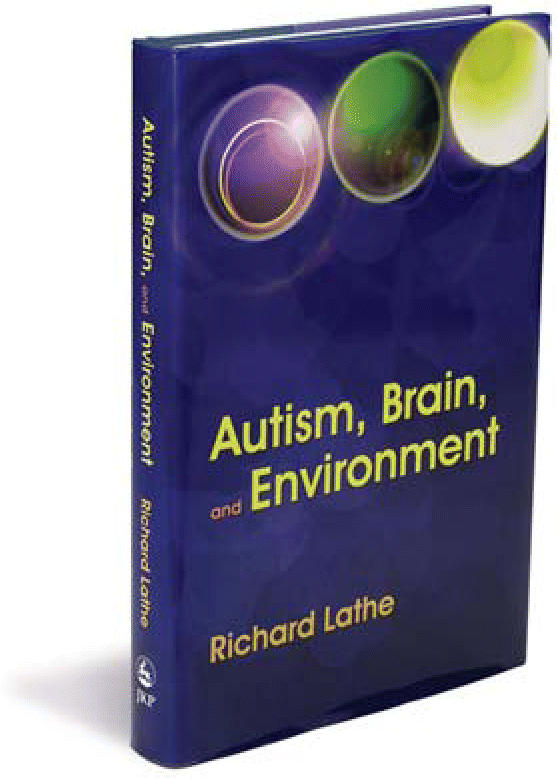# Autism, Brain, and Environment

**Published:** 2006-12

**Authors:** Gayle C. Windham

**Affiliations:** Gayle C. Windham is a research scientist/epidemiologist in the Environmental Health Investigations Branch of the California Department of Health Services and is an investigator in the California Center for Developmental Disabilities and Autism Research and Epidemiology (CADDRE). Her research focuses on the effects of environmental exposures on reproductive and developmental outcomes

By Richard Lathe

London:Jessica Kingsley Publishers, 2006. 288 pp. ISBN: 1-84310-438-5, $24.95

Autism rates have risen to “epidemic” proportions, as we hear from media reports. Yet understanding why has so far been elusive, and a myriad of theories have been proposed, from changing diagnostic criteria to increased awareness to vaccines to different mating patterns increasing the likelihood of familial inheritance. In this book, Richard Lathe takes a scholarly approach to exploring a variety of possible links in order to explain autism, pulling together evidence from numerous fields of study including, among others, neuroscience, toxicology, genetics, endocrinology, and immunology.

Autism or autistic spectrum disorder (ASD) is a devasting disability, with lifelong implications for all but those only mildly affected. Thought to be rare when first described in the 1940s, it is now reported to occur in 1 of every 166 births. There is no medical test for autism, but its diagnosis is based on behavioral impairments in three areas: communication, social interactions, and repetitive or restricted activities. Lathe has written a very thorough and clear book describing autism, integrating the evidence that has led him to conclude that it is a disorder of the limbic brain, which is very sensitive to environmental toxicants, while recognizing the genetic contribution as well.

Lathe describes his intent to provide material accessible to researchers as well as nonspecialists, including families, medical practitioners, teachers, psychologists, and advocates, and he has done so to a reasonable extent. Each chapter begins with a simple, interesting introduction and ends with 5–10 key take-home points. The chapter material is clearly laid out, but of a fairly technical nature for the most part. Each chapter is extensively referenced with the latest literature. Thus the book provides a wealth of material to new researchers in the field and is bound to provide something of interest to experienced investigators because of the breadth covered. Some of this evidence is not very critically reviewed, so that, for example, one study on a topic is offered as conclusive evidence at times. But readers can look at the original references to draw their own conclusions.

Lathe came not from the field of autism, but rather neuroscience. On meeting an affected child, he was struck by how autistic behavior resembled that of people with injuries to specific areas of the brain—the hippocampus and amygdala—which guided his investigation. The book begins with a description of ASD and its diagnosis, and then covers the strong genetic component, even touching on epigenetics, and concluding that a “two-hit” mechanism is likely—genetic susceptibility and exposure to an environmental factor. After reviewing the evidence for increased rates, Lathe concludes that the rise in number of ASD cases may be real; he terms this recent trend “new phase autism,” which involves a much stronger environmental component than classic autism. Whereas most autism researchers would agree that the rate has increased, but perhaps not why or how much, Lathe pragmatically suggests that the reasons may not matter at this point—ASDs are of such major concern that they demand understanding regardless.

Environmental factors are explored with a strong emphasis on heavy metals and brain function; although some conflicting evidence is oversimplified, Lathe does avoid fanning so far unsubstantiated concerns about vaccines. He touches on the roles of immune and hormone factors, but only lightly. The numerous other physiologic problems diagnosed in children with ASD are described, with the conclusion that far from being separate from the psychiatric aspects, these physiologic problems may produce and exacerbate the condition. Subtyping by these or genetic factors may focus research to elucidate specific mechanisms and lead to the most efficacious biochemical treatments or prevention. Lathe finishes by taking a wider of view of autism, drawing parallels to other multifactorial conditions, such as Alzheimer disease.

## Figures and Tables

**Figure:**